# Estimating the re-identification risk of clinical data sets

**DOI:** 10.1186/1472-6947-12-66

**Published:** 2012-07-09

**Authors:** Fida Kamal Dankar, Khaled El Emam, Angelica Neisa, Tyson Roffey

**Affiliations:** 1 Children’s Hospital of Eastern Ontario Research Institute, Ottawa, ON, K1H 8L1, Canada; 2 Pediatrics, University of Ottawa, Ottawa, ON K1H 8L1, Canada; 3 Children’s Hospital of Eastern Ontario, Ottawa, ON K1H 8L1, Canada; 4 CHEO Research Institute, 401 Smyth Road, Ottawa, ON K1H 8L1, Canada

## Abstract

**Background:**

De-identification is a common way to protect patient privacy when disclosing clinical data for secondary purposes, such as research. One type of attack that de-identification protects against is linking the disclosed patient data with public and semi-public registries. Uniqueness is a commonly used measure of re-identification risk under this attack. If uniqueness can be measured accurately then the risk from this kind of attack can be managed. In practice, it is often not possible to measure uniqueness directly, therefore it must be estimated.

**Methods:**

We evaluated the accuracy of uniqueness estimators on clinically relevant data sets. Four candidate estimators were identified because they were evaluated in the past and found to have good accuracy or because they were new and not evaluated comparatively before: the Zayatz estimator, slide negative binomial estimator, Pitman’s estimator, and mu-argus. A Monte Carlo simulation was performed to evaluate the uniqueness estimators on six clinically relevant data sets. We varied the sampling fraction and the uniqueness in the population (the value being estimated). The median relative error and inter-quartile range of the uniqueness estimates was measured across 1000 runs.

**Results:**

There was no single estimator that performed well across all of the conditions. We developed a decision rule which selected between the Pitman, slide negative binomial and Zayatz estimators depending on the sampling fraction and the difference between estimates. This decision rule had the best consistent median relative error across multiple conditions and data sets.

**Conclusion:**

This study identified an accurate decision rule that can be used by health privacy researchers and disclosure control professionals to estimate uniqueness in clinical data sets. The decision rule provides a reliable way to measure re-identification risk.

## Background

The public is uncomfortable disclosing their personal information, or having their personal information processed for, secondary purposes if they do not trust the organization collecting and processing the data. For example, individuals often cite privacy and confidentiality concerns and lack of trust in researchers as reasons for not having their health information used for research purposes [1]. One study found that the greatest predictor of patients’ willingness to share information with researchers was the level of trust they placed in the researchers themselves [2]. A number of US studies have shown that attitudes toward privacy and confidentiality of the census are predictive of people’s participation [3,4], and also that there is a positive association between belief in the confidentiality of census records and the level of trust one has in the government [5]. These trust effects are amplified when the information collected is of a sensitive nature [5,6].

There is a risk that the increasing number of medical data breaches are potentially eroding the public’s trust in health information custodians in general [7,8]. For example, the number of records affected by breaches is already quite high: the U.S. Department of Health and Human Services (HHS) has reported 252 breaches at health information custodians (e.g., clinics and hospitals) each involving more than 500 records from the end of September 2009 to the end of 2010 [9]. In all, the records of over 7.8 million patients have been exposed. At the same time there is increasing pressure to make individual-level health data more generally available, and in some cases publicly available, for research and policy purposes [10-23].

One of the factors which help to make the public more comfortable with their health information being used for research purposes is its de-identification at the earliest opportunity [1,24-30]. As many as 86% of respondents in one study were comfortable with the creation and use of a health database of de-identified information for research purposes, whereas only 35% were comfortable with such a database that included identifiable information [28]. It is therefore important to ensure that the risk of re-identification is low.

The uniqueness of individuals in the population is often used as a measure of re-identification risk [31-36]. In commentary in the Federal Register about the de-identification standards in the Health Insurance Portability and Accountability Act (HIPAA), HHS referred only to uniqueness as the re-identification risk measure [37,38]. If an individual is unique in the population then their risk of re-identification can be quite high. For example, unique individuals are easier to correctly re-identify by matching their records in the disclosed database with a population registry, such as a voter registration list [39].

When the data custodian is disclosing the full population of patients then it is easy to just measure uniqueness from the data. However, in practice many data sets are samples from the population, for example, data abstracted from a sample of charts, data from surveys [40,41], and public use microdata files such as census sample files [42-46]. The population may be all of the patients at a clinic or all people living in a particular geographic area.

The custodian may not have the resources to acquire data on all of the population to measure re-identification risk [47]. Consequently, the custodian needs to estimate uniqueness from the available sample data, and then decide whether the risk of re-identification is acceptable or if further disclosure control actions are required (e.g., generalization of the data or putting in place a data sharing agreement with the data recipient).

A number of different uniqueness estimators have been proposed in the literature. It is important to know which of these works best on clinical data sets. However, many of these estimators have not been compared, and therefore we do not know which ones would provide the most accurate estimates. In this study we use a Monte Carlo simulation to compare four different methods for estimating population uniqueness to determine which is the most accurate, and under what conditions.

## Methods

### Definitions

#### Quasi-identifiers

The variables that are going to be included in a risk assessment are called the *quasi-identifiers*[48]. Examples of common quasi-identifiers are [33,49-52]: dates (such as, birth, death, admission, discharge, visit, and specimen collection), locations (such as, postal codes, hospital names, and regions), race, ethnicity, languages spoken, aboriginal status, and gender.

#### Equivalence classes

All the records that have the same values on the quasi-identifiers are called an *equivalence class*. For example, all the records in a dataset about 17 year old males admitted on 1^st^ January 2008 are an equivalence class.

#### Uniqueness

A unique record is one that is in an equivalence class of size one. For example, if our quasi-identifiers are age, gender, and postal code, then if there is only one 90 year old female in the postal code “N3E 6Y4” then her record would be unique. Other sensitive variables that are not considered quasi-identifiers are not taken into account in the computation of uniqueness. The term “uniqueness” is used to characterize the amount of unique records in a data set. The way it is measured will depend on other factors, and these are discussed further below.

### Threat model and risk measurement

#### Context

Consider the common situation whereby a data custodian wishes to disclose a data set to a researcher. A condition of the disclosure by the research ethics board was that the data has to be de-identified. To decide whether the data set is sufficiently de-identified, the data custodian needs to measure re-identification risk.

One of the common threat models that is considered when disclosing health data sets is that an adversary will match against the voter registration list [39], and in the responses to comments on the HIPAA Privacy Rule regulations published in the Federal Register, the Department of Health and Human Services (DHHS) explicitly considers voter registration lists as a key data source that can be used for re-identification [37,38]. Some legal scholars argue that threat models should only consider *public* information which an adversary can get access to and not information that may be privately known by the adversary or in private databases [53].

**Figure 1:**
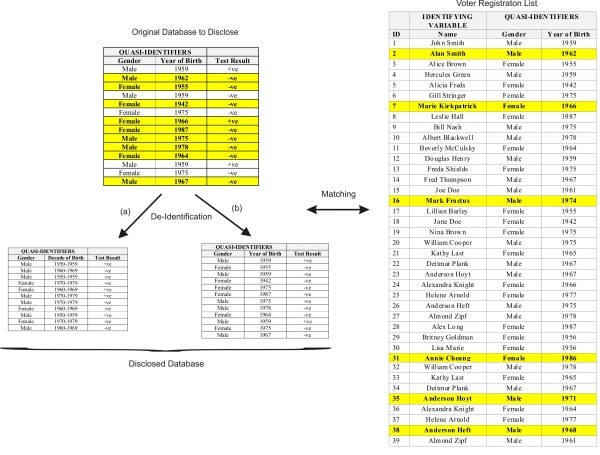
Example of a data set to be disclosed and how uniqueness makes it easier to re-identify individuals by matching to a voter list.

The voter registration list is assumed to represent the whole adult population. Many states in the US make their voter registration lists readily available for a nominal fee or free, and these often include the name, address, date of birth, and gender of individuals [39]. The matching example is shown in Figure 1.

Under this example the data that is being disclosed is considered a sample, and the voter registration list is considered the population. In our analysis we assume that the adversary does not know who is in the sample data set. For instance, the sample may be charts randomly selected for abstraction.

Here we have 14 individuals in the sample data set. An examination of that data set indicates that 9 of the 14 records are unique on the quasi-identifiers (they are highlighted in the figure). Given that they are unique in the data set, then the custodian may assume that if an adversary links these records with the voter list they will all match successfully and all 9 can be re-identified: a re-identification rate of approximately 64%, which would be considered high by most standards. The data custodian may then proceed to generalize the year of birth to a decade of birth such that none of the records in the data set is unique and suppresses three records in the data set (approximately 21% suppression). This is illustrated in de-identification path (a) in Figure 1. By eliminating uniqueness the adversary would not be able to match with certainty any of the disclosed records. This de-identification has resulted in the loss in precision of the date of birth variable and 21% suppression.

However, the data custodian did not need to generalize the year of birth at all. For a correct match to occur *with certainty*, a record needs to be unique in *both*, the disclosed data set as well as in the voter registration list. As shown in Figure 1, only 2 of the 9 records that are unique in the original data set are also unique in the voter registration list (the unique records in the voter registration list are highlighted). Therefore, under our threat model the data custodian could have disclosed the original data with the full year of birth and only suppressed these two records (the male born in 1962 and the female born in 1966). This is illustrated in de-identification path (b) in Figure 1. We are only interested in the records that are unique in the population given that they are unique in the sample data set.

#### Notation

We will first introduce some notation. Let *N* and *n* be the number of records in the voter registration list and the disclosed (sample) data set respectively, *K* and *u*denote the number of non-zero equivalence classes in the voter registration list and the disclosed data set respectively, and $F_{i}$ and $f_{i}$ denote the size of the *i*^th^ equivalence class in the voter registration list and the disclosed data set respectively, where $i\in \left\{1,\ldots K\right\}$ ($\left\{1,\ldots ,u\right\}$ respectively).

#### Measuring uniqueness

One can measure the conditional probability that a record in the voter registration list is unique given that it is unique in the original data set by [54]:

(1) $$\lambda_{1}=\frac{\sum_{i}I\left(f_{i}=1,F_{i}=1\right)}{\sum_{i}I\left(f_{i}=1\right)}$$

where *I* is the indicator function. For example, $I\left(f_{i}=1,F_{i}=1\right)$ is one if the sample equivalence class is a unique as well as the corresponding population equivalence class, otherwise it is zero.

However, as a risk metric for the whole data set that will be disclosed, $\lambda_{1}$ can be misleading. In our example, 2 out of 9 sample unique records were population unique, giving a risk of $\lambda_{1}=0.22$. However, out of the whole data set only 2 out of 14 records are at risk, therefore the data set risk should be 0.14. To give a more extreme example, consider a 1000 record data set where there are only two unique records and they are both also unique in the voter registration list. In this case $\lambda_{1}=1$ indicating that *all* records are at risk, when in fact only 2 out of 1000 records are at risk. A more appropriate risk metric would then be:

(2) $$\lambda_{2}=\frac{\sum_{i}I\left(f_{i}=1,F_{i}=1\right)}{n}$$

In the 1000 record example above, this would give a risk of $\lambda_{2}=0.002$ and for the example of Figure 1 it would be $\lambda_{2}=0.14$ for the original data set, which corresponds to what one would expect intuitively.

The risk metric $\lambda_{2}$ approximates the proportion of records in the voter registration list that are unique under an assumption of sampling with equal probabilities [54]. The $\lambda_{3}$ measure is the proportion of records in the voter registration list that are unique:

(3) $$\lambda_{3}=\frac{\sum_{i}I\left(F_{i}=1\right)}{N}$$

The value for $\lambda_{3}$ in our example of Figure 1 would be 0.15 since six records in the voter registration list are unique.

**Figure 2:**
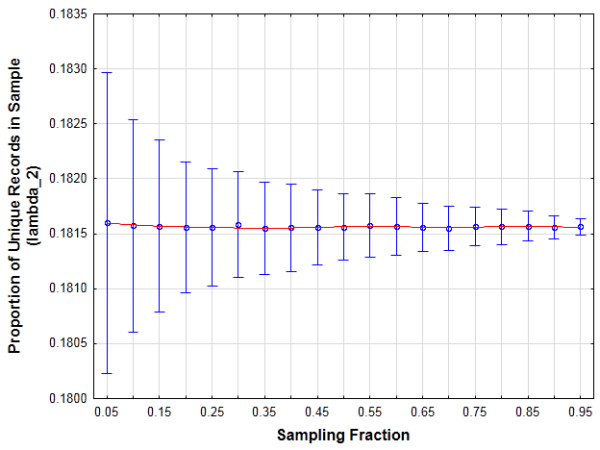
**The mean proportion of unique records in samples drawn from the NY State Inpatient Database data set for 1000 samples at different sampling fractions for month/year of birth, ZIP3, gender, length of stay in days, and time since last visit in days. **The whiskers represent the standard deviation. The population risk value is 0.1815.

To illustrate the relationship between the measures in equations (2) and (3), we empirically computed the expected value $E\left(\lambda_{2}\right)$ on the state inpatient database for the state of New York for 2007. This data set, which is available from the Agency for Healthcare and Quality, consists of discharge abstract data for approximately 1.5 million patients (after removing patients with invalid ZIP codes). We used the following quasi-identifiers: age in years, gender, the first three digits of the ZIP code, the time in days since the last visit, and the length of stay at the hospital in days. In the whole population 0.1815 of the records were unique (i.e., $\lambda_{3}=0.1815$). We drew 1000 random samples at varying sampling fractions from that population data set and computed the mean $\lambda_{2}$. As you can see in Figure 2, the $E\left(\lambda_{2}\right)$ value is very close to the $\lambda_{3}$ value across sampling fractions.

Therefore, if we can compute or estimate $\lambda_{3}$ directly, then we would get a measure of risk for any sample data set under an assumption of sampling with equal probabilities. This metric would have an intuitive general meaning.

There is evidence in the responses to commentary on HIPAA in the Federal Register by DHHS that they were thinking of $\lambda_{3}$ as the re-identification risk metric in the discussion of identifiability, for example, when there is reference to "At the point of approximately 100,000 population, 7.3% of records are unique" and "4% unique records using the 6 variables", which in all cases were based on analyses of census data and in all cases was referring to the percentage of all records in the file [37,38]. Furthermore, the actual re-identification risk of data sets compliant with the HIPAA Safe Harbor standard has been computed empirically and is always presented in terms of a $\lambda_{3}$ metric [55-57].

To know in advance the proportion of records in the voter registration list that are unique, the data custodian has two options: (a) obtain a copy of the voter registration list for all areas of the country for which there are patients in the data set and compute the number of records that are unique in the voter registration list on the quasi-identifiers, or (b) estimate uniqueness in the voter registration list using the disclosed data set only. The former can be resource intensive and would require regularly acquiring an updated voter list. The latter is less costly and can be fully automated.

Our objective in this paper then is to evaluate existing uniqueness estimators of the form $\lambda_{3}$ and identify one or a combination of estimators that are most accurate. The data custodian can use the estimator with only the disclosed data set to assess re-identification risk. If that number is too high then the custodian can apply various de-identification methods, such as generalization and suppression, to reduce it to an acceptable level. The steps of such a process are described later in the paper.

### Estimating uniqueness

Thus far there have been no comprehensive evaluations of existing uniqueness estimators of the type $\lambda_{3}$. In this study we will empirically evaluate a set of population uniqueness estimators to determine which ones provide the most accurate estimates.

Various models were used in the literature to estimate the population uniqueness from a sample. The majority are based on the superpopulation model approach. This approach assumes that the population is generated from a superpopulation by an appropriate distribution. The problem of population uniqueness estimation then becomes a problem of parameter estimation. The superpopulation methods proposed in the literature are: the Poisson-gamma model [31], the Poisson lognormal model [58], the Logarithmic series model [59], the Dirichlet multinomial model [60], the Ewens model [61], Pitman’s model [62,63], and the slide negative binomial model [64]. The mu-argus model [65] has not been used in the context of population uniqueness estimation, but can be extended for that purpose. Furthermore, Zayatz introduced a method which is not dependant on a model for the population equivalence classes [66].

Hoshino [63] compared 6 superpopulation models: the Poisson-gamma model, the Poisson lognormal model, the Logarithmic series model, the Dirichlet multinomial model, the Ewens model, and Pitman’s model. He concluded that the Pitman model “provides the most plausible inference” among the models compared. Based on his comparison, we will discard the 5 models above since they were inferior in estimation accuracy, and include only the Pitman model in our evaluation.

[Chen and McNulty 64] compared 3 models: the slide negative binomial (SNB) model, the equivalence class model and the Poisson-gamma model. They concluded that the SNB model improves significantly the population uniqueness estimation. However, the authors assumed that the number of equivalence classes in the population is known and they employed that fact in assessing the models. In practice however, the number of population equivalence classes is not known (and must also be estimated), and for that reason these results are not realistic. It is necessary to re-run that comparison and therefore we will include the SNB model and the Zayatz equivalence class model in our evaluation.

In this paper we therefore evaluate the following four models: [Zayatz 66], SNB [64], the Pitman model [62,63], and mu-argus [65]. Based on existing evidence, these models are the best candidates for estimating uniqueness and have not been compared directly on clinical data sets before.

### Empirical evaluation

#### Simulation

We performed a Monte Carlo simulation to evaluate the accuracy of the four estimators described above. In this simulation we mimic what the adversary would do and therefore we mimic the re-identification success rate of the adversary. We assume that a disclosed data set is a subset from a population data set. An adversary will match the records in the disclosed data set with the population (as explained in our motivating example). The number of records that can be matched with certainty is on average equal to $\lambda_{3}$. We could compute $\lambda_{3}$ exactly from the population data set. This gave us the actual re-identification success rate of the adversary.

All estimators were implemented by the authors in SAS, and all simulations described here were also performed in SAS. The estimators and the parameter choices, where relevant, are described further in the Additional file 1: Appendix A.

#### Data sets

The six data sets we used are shown in Table 1. The first three are public and last three are confidential clinical data sets. They all have the typical kinds of demographic quasi-identifiers that are seen in clinical data sets. These data sets were chosen because of their heterogeneity – since they represent different types of contexts they increase the generalizability of the results.

**Table 1:** The data sets that will be included in our simulation

**Description**	**Quasi-identifiers**	**No. Records**
**Adult**		32,561
The adult dataset from the UC Irvine machine learning data repository. This is an extract from the US census and has common demographics and socio-economic status variables: ftp://ftp.ics.uci.edu/pub/machine-learning-databases/adult	· Age	
	· Profession	
	· Education	
	· Marital status	
	· Race	
	· Sex	
	· Country	
**FARS**	·	43,330
Department of Transportation Fatal crash information: http://www-fars.nhtsa.dot.gov/main.cfm	· Age	
	· Race	
	· Month of Death	
	· Day of Death	
**CUP**		95,412
Data from the Paralyzed Veterans Association on veterans with spinal cord injuries or disease: http://kdd.ics.uci.edu/databases/kddcup98/kddcup98.html	· ZIP code	
	· Age	
	· Gender	
	· Income	
**Pharm**		16,424
Prescription records from the Children’s Hospital of Eastern Ontario pharmacy from July 2006 to March 2009. This is for inpatients only and excludes acute cases. A de-identified version of this data was disclosed to commercial data aggregators [67].	· Age	
	· Postal code (FSA)	
	· Admission date	
	· Discharge date	
	· Sex	
**ED**		108,344
Emergency department records from Children’s Hospital of Eastern Ontario from 1^st^ June 2007 to 1^st^ June 2009. This data is disclosed for the purpose of disease outbreak surveillance.	· Admission date	
	· Postal Code	
	· Date of Birth	
	· Sex	
**Niday**		637,964
A registry of all newborns in Ontario from 1^st^ April 2004 to 31^st^ March 2009. This data set is used frequently for research purposes: http://www.bornontario.ca	· Maternal postal code	
	· Baby DoB	
	· Mother DoB	
	· Baby sex	

Each data set is treated as a population. The data set size as well as the variables which will be included in the analysis are shown.

Three different versions of each data set were created, with low uniqueness (<10% of the observations), medium uniqueness (between 10% and 50% of the observations), and high uniqueness (greater than 50% of the observations). The three versions of the data sets were created by generalizing the quasi-identifiers in the original data set. For example, a date of birth may be generalized to year of birth, or a six character postal code may be generalized to a three character postal code. The FARS and Adult data sets only had medium uniqueness at the outset, therefore there was no possibility of creating a high uniqueness version of these data sets.

#### Measurement

We treat each data set as a population and draw 1000 simple random samples. For each sampling fraction we compute the median relative bias across the 1000 samples: medianλ^3−λ3λ3. We also compute the inter-quartile range which indicates the dispersion of the relative bias.

The relative bias is suited to this problem because it reflects the importance of the error in decision making better than, say, just the bias $\left({\hat{\lambda }}_{3}-\lambda_{3}\right)$. Because the most common acceptable values for uniqueness are often low (for example, between 0.05 and 0.2 [68-70]), the bias can give misleading results. For example, a bias of 0.1 when $\lambda_{3}=0.9$ is not going to influence the decision that the re-identification risk is high. However, a bias of 0.1 when $\lambda_{3}=0.11$ could make a difference in deciding whether the risk is acceptable or not. In both cases the bias is the same, but the impact on the decision is quite different. The relative bias, on the other hand, would be quite low in the former case (0.11), and high in the latter (0.91), which more accurately reflects the severity of the error.

An alternative evaluation metric that could have been used was a mean square error (MSE). However, extreme values for some of the estimators under some simulation conditions distorted the MSE significantly. Hence, we chose a robust median to get a more realistic assessment of performance.

#### Model combination

Three parameters were varied during this simulation: (a) the data set used to represent the population, (b) the extent of uniqueness in the population, and (c) the sampling fraction.

The sampling fraction was varied for each data set as follows: 0.01, 0.05, 0.1, 0.3, 0.5, 0.7, and 0.9. In total then, there were 3 (uniqueness levels) x 7 (sampling fractions) x 4 (estimators) = 84 study points per data set simulated 1000 times.

Informed by methods to create ensembles [71,72], we combined the estimators that we have to try to obtain a more accurate estimate that utilizes as many of our base estimation methods as possible. A simple ensemble would take the mean of the estimates of all of the estimators. However. we expected that some estimators will work better under different conditions (e.g., for different values on sampling fraction or population uniqueness value), and we wanted our ensemble strategy to take that into account.

We therefore constructed a regression tree across all study points for each data set [73]. The outcome variable used when constructing the tree was the relative bias results for each observation (where there are 84,000 observations). A regression tree provides a succinct descriptive summary of the factors that affect estimation accuracy and can be helpful in discovering subtle patterns. The input variables for constructing the tree were the sampling fraction, the estimator, and the uniqueness level. The tree construction process attempts to reduce the node deviance, defined as $\sum {\left(y-\bar{y}\right)}^{2}$, where *y* is the relative bias and $\bar{y}$ is the mean relative bias within a node.

Because ensembles are usually created for a single data set, we had six trees. We then used a subjective process to combine the regression trees from each data set to create an overall decision rule. In developing this decision rule we assumed that under-estimation is worse than over-estimation. Under-estimation may result in a data custodian inadvertently disclosing data with a high amount of uniqueness, and therefore exposing patient data to a higher re-identification risk than intended. Over-estimation leads to a conservative approach to disclosure where data that has been disclosed has a lower re-identification risk than intended.

### Ethics

This study was approved by the research ethics board of the Children’s Hospital of Eastern Ontario. The data custodians for the three non-public clinical data sets also approved this protocol.

## Results

We present the detailed results for the emergency department data set in the main body of the paper, with the results for the other data sets in the Additional file 2: Appendix B. The results were quite consistent across the data sets and therefore here is no loss in generality by focusing on the emergency department data here.

**Figure 3:**
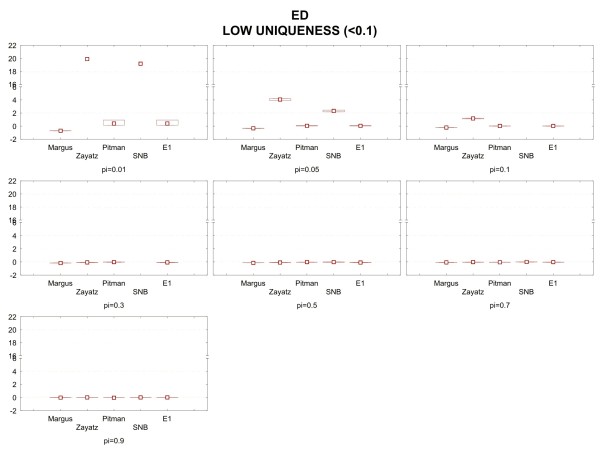
Median relative bias and inter-quartile range results under the low uniqueness condition for the emergency department data.

Figure 3 shows the median relative bias and interquartile ranges of the relative bias for the emergency department data when the population uniqueness is below 10%. Each panel in the figure is for a particular sampling fraction (denoted by pi), and shows the results for the four estimators. We see that at low sampling fractions the models tend to have higher relative bias, and that approaches zero as the sampling fraction increases. Also, the amount of variation in the relative bias is not high.

**Figure 4:**
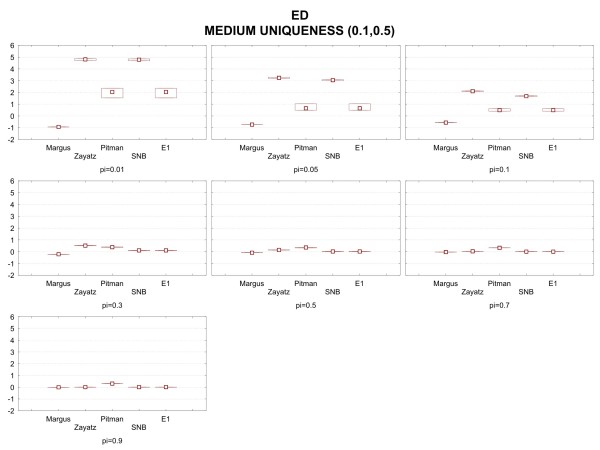
Median relative bias and inter-quartile range results under the medium uniqueness condition for the emergency department data.

In Figure 4 are the results (the median relative bias and interquartile ranges of the relative bias) when the population uniqueness is at a medium level (between 10% and 50%). The general pattern seen for low uniqueness holds, except there are a number of study points for which the SNB model fails. Also, the median relative bias is lower for all sampling fractions compared to the low uniqueness version of the data set.

**Figure 5:**
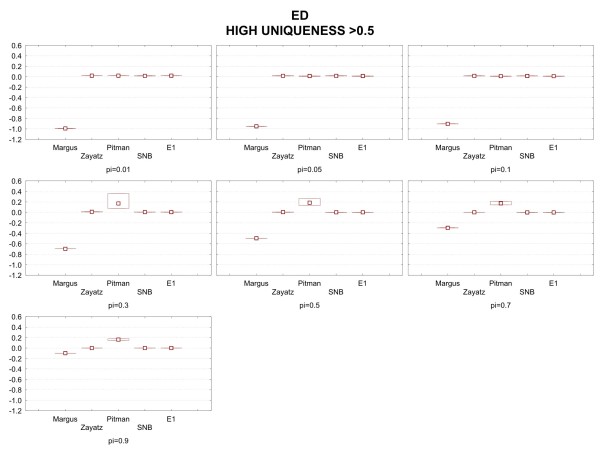
Median relative bias and inter-quartile range results under the high uniqueness condition for the emergency department data.

Figure 5 shows the results when there is high uniqueness in the population data set (greater than 50%). All models perform relatively well in terms of relative bias and variation of relative bias. This is the case even for small sampling fractions.

**Figure 6:**
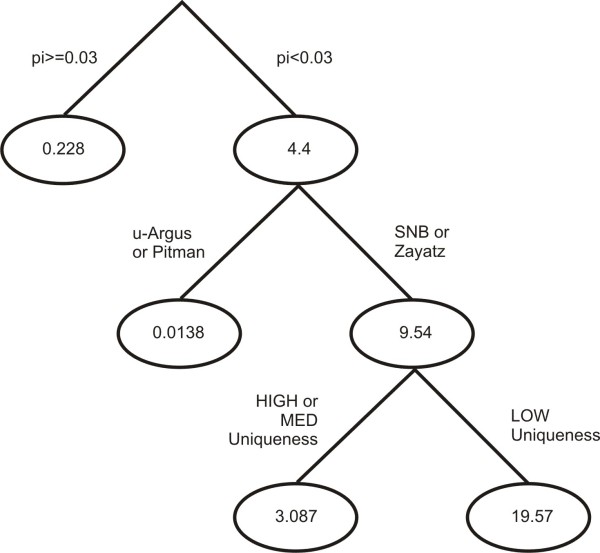
**The regression tree for the emergency department data set constructed from the 84,000 simulation results. **The numbers in the nodes are the mean relative error values.

The regression tree for the emergency department data is given in Figure 6. This shows that for higher sampling fractions (denoted by pi) all models tend to perform well with a mean relative bias of 0.22. For lower sampling fractions the Pitman model and the mu-Argus model have the lowest mean relative bias at 0.013. When the sampling fraction is low (below 30%) the SNB and Zayatz models tend to have high relative bias, irrespective of the uniqueness levels in the data.

In general we found that the Pitman model emerged as the most accurate for low sampling fractions. For higher sampling fractions the most accurate estimate varies between SNB and Zayatz. However, SNB tended to fail to converge in a number of instances, making it an unreliable model in practice and required us to have a 'replacement' in our decision rule.

The combined rule from the six data set ensembles is shown below. The performance of that rule compared to the original models is given in the results graphs in Figures 3, 4, and 5 and is labeled as the E1 model. As can be seen, the performance of E1 is superior to any of the original models across the full set of conditions.

If π ≤ 0.1 then

E1 = Pitman

Else

If SNB converges then

if Est(SNB) > Est(Zayatz) then

E1 = Zayatz

Else

E1 = SNB

Endif

Else

E1 = Zayatz

Endif

Endif

The E1 rule does not use the mu-argus estimator. The mu-argus estimator consistently performed worse than the other estimators and was associated with terminal nodes with high relative bias in all of the regression tree. Therefore its inclusion would have resulted in a noticeable deterioration in prediction performance.

## Discussion

### Summary and implications

Population uniqueness is a commonly used measure of re-identification risk [31-36]. In cases where the disclosed data set is a sample, the population uniqueness must be estimated. In this paper we have evaluated four different uniqueness estimators using a Monte Carlo simulation on clinically relevant data sets.

Informed by methods to creating ensembles, we constructed regression trees that combine the uniqueness estimators to minimize their relative bias for each data set. These trees were then converted to a single decision rule that works across all data sets and performs better than any of the original estimators.

Our decision rule selects among the best three estimators. It has good and consistent accuracy across multiple conditions, often with a small overestimation. Application of the decision rule requires the implementation of three estimators. However, it does not require knowledge of the general uniqueness level in the population a priori (i.e., if it is low, medium, or high), which may be difficult to know in practice, but does require knowledge of whether the sampling fraction is greater than 10% or not.

Future studies that need to estimate uniqueness should consider using the three estimators combined with this decision rule for maximum accuracy.

### Applications in practice

**Figure 7:**
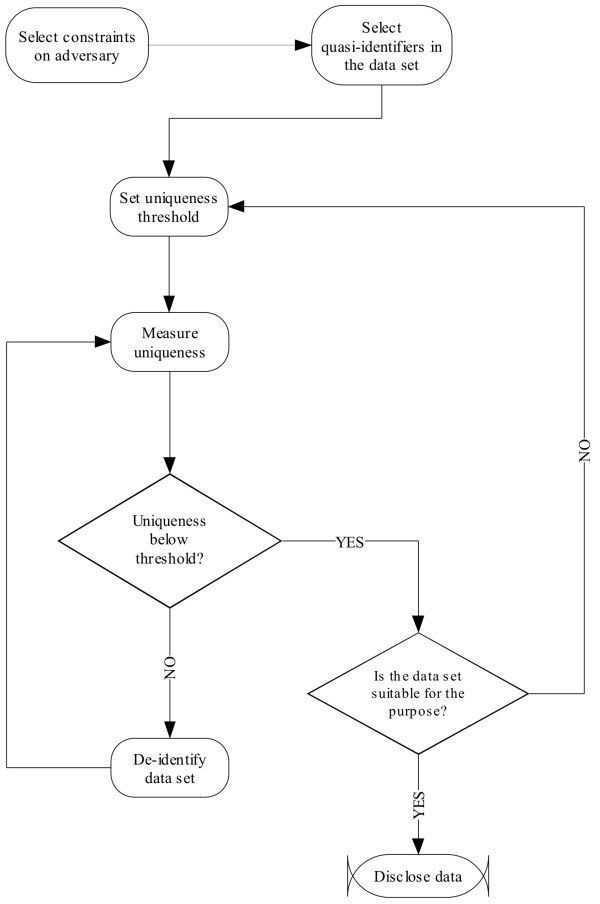
The process steps to apply the uniqueness estimators in practice.

The process within which uniqueness estimates would be applied is illustrated by the control flow graph in Figure 7.

The first step is for the custodian to understand the plausible adversaries that can attempt to re-identify the disclosed data. A useful way to categorize adversaries is in terms of how constrained they are. Five types of constraints to be considered are:

· **Financial constraints:** how much money will the adversary spend on a re-identification attack ? Costs will be incurred to acquire databases. For example, the construction of a single profession-specific database using semi-public registries that can be used for re-identification attacks in Canada costs between $150,000 to $188,000 [49]. In the US, the cost for the voter registration list from Alabama is more than $28,000, $5,000 for Louisiana, more than $8,000 for New Hampshire, $12,000 for Wisconsin and $17,000 for West Virginia [39].

· **Time constraints:** how much time will the adversary spend to acquire registries useful for a re-identification attack? For example, let’s say that one of the registries that the adversary would use is the discharge abstract database from hospitals. Forty eight states collect data on inpatients [74], and 26 states make their state inpatient databases (SIDs) available through the Agency for Healthcare Research and Quality (AHRQ) [75]. The SIDs for the remaining states would also be available directly from each individual state but the process may be more complicated and time consuming in this example. Would an adversary satisfy themselves only with the AHRQ states or will they put the time to get the data from other states as well ?

· **Willingness to misrepresent themselves:** to what extent will the adversary be willing to misrepresent themselves to get access to public or semi-public registries? For example, some states only make their voter registration lists available to political parties or candidates (e.g., California) [39]. Would an adversary be willing to misrepresent themselves to get these lists? Also, some registries are available at a lower cost for academic use versus commercial use. Would a non-academic adversary misrepresent themselves as an academic to reduce their registry acquisition costs?

· **Willingness to violate agreements:** to what extent would the adversary be willing to violate data sharing agreements or other contracts that s/he needs to sign to get access to registries? For example, acquiring the SIDs through the AHRQ requires that the recipient sign a data sharing agreement which prohibits re-identification attempts. Would the adversary still attempt a re-identification even after signing such an agreement?

· **Willingness to commit illegal acts:** to what extent would an adversary break the law to obtain access to registries that can be used for re-identification? For example, privacy legislation and the Elections Act in Canada restrict the use of voter lists to running and supporting election activities [49]. There is at least one known case where a charity allegedly supporting a terrorist group has been able to obtain Canadian voter lists through deception for fund raising purposes [76-78].

It should be noted that most known re-identification attacks were performed by researchers or the media [79]. This type of adversary is likely highly constrained with limited time and funds, an unwillingness to misrepresent themselves, and unwillingness to violate agreements and contracts. Alternatively, the custodian may wish to make a worse case assumption and consider a minimally constrained adversary with unlimited resources and funds who is willing to misrepresent themselves and violate agreements and laws. This kind of assumption would be suitable if the data will be made publicly available, in which case the data custodian would have no control over who would get the data. The choice of constraints will have an impact on which registries the adversary would plausibly have access to.

The data custodian then needs to select the quasi-identifiers in the data set. The quasi-identifiers would be the variables that a potential adversary would be able to get using public or semi-public registries. Note that an adversary may combine multiple sources together to construct a database useful for re-identification [50]. It is not necessary for the custodian to acquire all of these registries, but only to know what the variables are in these registries. Examples of public and semi-public registries that can be used for re-identification are:

· Voter registration lists, court records, obituaries published in newspapers or on-line, telephone directories, private property security registries, land registries, and registries of donations to political parties (which often include at least full address).

· Professional and sports associations often post information about their members and teams (e.g., lists of lawyers, doctors, engineers, and teachers with their basic demographics, and information about sports teams with their demographics, height, weight and other physical and performance characteristics).

· Certain employers often post information about their staff on-line, for example, at educational and research establishments and at law firms.

For a registry to be useful as a potential source of quasi-identifiers, it must be plausible for the adversary to get access to it. By considering the constraints on the adversary, it is then possible to decide how plausible it is for the adversary to acquire each type of registry and for which state. For example, if the data to be disclosed is for patients in California and it is assumed that the adversary is highly constrained, then the voter registration lists would not be available to the adversary for a re-identification attack (it is only available for parties, candidates, political committees, scholarly or journalistic purposes).

Because the assumptions made about the adversary would often not apply to the data custodian, it is important for the data custodian to be able to estimate re-identification risk. For example, if it is assumed that the adversary is willing to misrepresent themselves to get a semi-public registry, the data custodian cannot mimic that and misrepresent themselves to acquire that registry for the purpose of re-identification risk assessment. The custodian needs to estimate the risk without acquiring that registry, which is the problem our uniqueness estimators are solving.

The custodian must then select the uniqueness threshold that will be used to decide whether the re-identification risk is acceptable or not. There are a number of precedents that can be useful for deciding on a threshold. One can, for instance, rely on how HHS classifies health data breaches, whereby they will not publicize breaches affecting less than 500 records [80]. This effectively sets two tiers of breaches, and one can argue that a re-identification affecting less than 500 records would be considered lower risk. Also, previous disclosures of cancer registry data have deemed thresholds of 5% and 20% of the population at risk as acceptable for public release and research use respectively [68-70].

Now the data custodian can use the estimators and decision rule described in this paper to measure the actual uniqueness from the data using the selected quasi-identifiers. If the uniqueness estimate is larger than the threshold then the data custodian can de-identify the data by applying, for example, generalization and suppression [81]. If the uniqueness is below the threshold, then a decision needs to be made about whether the de-identified data is suitable for the purpose of the analysis that will be performed on it. This is a subjective decision that requires consultation with the data recipients. If the data is deemed not suitable for the purpose because there was too much generalization and suppression, then the threshold can be revised upwards.

Revising the threshold upwards implies that the data custodian is taking more risk in disclosing that data. To compensate for that higher risk, the custodian may wish to impose additional constraints or conditions. For example, the custodian may require that regular security audits be performed of the data recipient’s site. A systematic way for making these tradeoffs and the checklists that can be used for that purpose have been detailed elsewhere [35,82-84].

### Related work

An alternative mechanism for protecting information that has been proposed in the literature is differential privacy [85,86]. Generally speaking, differential privacy requires that the answer to any query be “probabilistically indistinguishable” with or without a particular row in the database. Thus differential privacy hides the presence of an individual in the database by making the two output distributions (with or without the row) “computationally indistinguishable” [87]. This is typically achieved by adding Laplace noise to every query output. The noise should be large enough in order to hide the output contributed by any row in the database. The literature on differential privacy, although extensive, has been mostly theoretical [86,88]. Moving from theory to practice will require specific limitations and considerations to be addressed [88], and it is proving to be a challenging task [89,90]. Therefore, for the context that we consider in this paper, the disclosure of individual-level data, differential privacy does not provide a ready solution yet, whereas managing uniqueness has been a generally accepted approach for disclosure control over the last two decades.

There are other criteria for deciding whether the risk of re-identification is too high. The most common is the k-anonymity criterion [91-94]. Uniqueness is the same as k-anonymity when k=1. If a data set has high uniqueness then it will fail the k-anonymity criterion for any value of k>1. If a data set has low uniqueness, then it may still fail k-anonymity for a higher value of k. Therefore, low uniqueness is a necessary but insufficient condition to achieve k-anonymity for k>1.

### Limitations

One assumption in our current threat model, and in almost all threat models used in the disclosure control literature, is that an adversary will use exact matching to re-identify individuals. In reality data sets have errors, duplicates, and other quality problems. Therefore, in general contemporary re-identification risk metrics tend to err on the conservative side.

We constructed a rule from six data sets. These were six data sets that were heterogeneous covering very different settings and were all clinically relevant in that they had quasi-identifiers often seen in clinical data sets and that could be used for re-identification. While it would be better to repeat the analysis on more data sets, we found considerable consistency in the trees generated from each data set. Furthermore, the final decision rule that we created performed well across all six heterogeneous data sets. Future work should further validate this rule on other independent data sets.

## Conclusions

Accurately measuring re-identification risk is necessary when using and disclosing health data for secondary purposes without patient consent. This allows the data custodian to ensure that patient privacy is protected in a defensible manner. Population uniqueness is a commonly used measure of re-identification risk. However, there are multiple methods for estimating population uniqueness that have been proposed in the literature, and their relative accuracy has not been evaluated on clinical data sets. In this study we performed a simulation to evaluate these estimation methods and based on that developed an accurate decision rule that can be used by health privacy researchers and disclosure control professionals to estimate uniqueness in clinical data sets. The decision rule provides a reliable way to measure re-identification risk.

## Competing interests

The author(s) declare that they have no competing interests.

## Authors’ contributions

FD designed the study, interpreted the results, and contributed to writing the paper. KEE designed the study, contributed to the data analysis, interpreted the results, and contributed to writing the paper. AN performed the data analysis and contributed to writing the paper. TR contributed to interpreting the results and writing the paper. All authors read and approved the final manuscript.

## Pre-publication history

The pre-publication history for this paper can be accessed here:

http://www.biomedcentral.com/1472-6947/12/66/prepub

## Supplementary Material

Additional file 1 **Appendix A. Uniqueness Estimation Models [**[63]**,**[65]**,**[95]**].** Click here for file

Additional file 2 **Appendix B. Detailed Results.** Click here for file
