# Supplementary material for: Estimating the re-identification risk of clinical data sets
Source: BMC Med Inform Decis Mak. 2012 Jul 9;12:66. doi: 10.1186/1472-6947-12-66 (PMC3583146; doi:10.1186/1472-6947-12-66)
Supplement: Additional file 1 — Appendix A. Uniqueness Estimation Models [[63],[65],[95]]. [file 1472-6947-12-66-S1.doc]

# Appendix A: Uniqueness Estimation Models

We will first introduce some notation then briefly describe all the estimation models evaluated in this paper. Let $N$ and $n$ be the size of the population and the sample respectively, $K$ and $u$denote the number of non-zero equivalence classes in the population and the sample respectively, and $F_{i}$ and $f_{i}$ denote the size of the $i$^th^ equivalence class in the population and the sample respectively, where $i\in\{1,...K\}$ ($\{1,...,u\}$ respectively). Also, let $S_{i}$ and $s_{i}$be the number of equivalence classes of size $i$ in the population and the sample respectively, and let $\pi$ be the sampling fraction.

We define $P(F=1|f=1)$as the probability that an equivalence class of size 1 in the sample was chosen from an equivalence class of size 1 in the population. Note, however, that estimating the number of sample uniques that are populaiton uniques does not tell us how by itself many uniques are in the population.

Below is a brief description of the models evaluated using the above notation:

- **Equivalence class model (Zayatz):** This model uses Baye’s rule to calculate the probability that a sample unique is also a population unique: $P(F=1|f=1)=\frac{p_{1}P(f=1|F=1)}{\sum_{j} p_{j}P(f=1|F=j)}$ where $p_{j}$ is the proportion of the equivalence classes in the population that are of size $j$, $p_{j}$ is estimated using the sample, and $P(f=i|F=j)$ follows a hypergeometric distribution for all values of $j$. The number of population uniques becomes: $S_{1}=s_{1}P(F=1|f=1)\frac{N}{n}$.
- **Pitman model:** the pitman model is defined by: $P(S_{1},S_{2},...,S_{N})=N!\frac{\theta(\theta+\alpha)(\theta+2\alpha)...(\theta+(K-1)\alpha)}{\theta(\theta+1)...(\theta+N-1)}\prod_{j=1}^{N} \left[\frac{(1-\alpha)^{[j-1]}}{j!}\right]^{S_{j}}\frac{1}{S_{j}!}$ where $(1-\alpha)^{[j-1]}=(1-\alpha)(2-\alpha)...(j-1-\alpha)$, and $\theta$ and $\alpha$ are real parameters describing the sampling scheme in the Pitman model (refer to [66] for more details). The population uniques are then estimated as: $E(S_{1})=\frac{\Gamma(\theta+1)}{\Gamma(\theta+\alpha)}N^{\alpha}$ where $\Gamma$ is the gamma function. The Pitman model has the exchangeability property with respect to individuals in the population. Hoshino uses this property to construct the Maximum Likelihood Estimators of $\theta$ and $\alpha$ [66]:

$$\begin{matrix} \frac{\partial L}{\partial\theta}=\sum_{i=1}^{u-1} \frac{1}{\theta+i\alpha}-\sum_{i=1}^{n-1} \frac{1}{\theta+i}=0 \\ \frac{\partial L}{\partial\alpha}=\sum_{i=1}^{u-1} \frac{i}{\theta+i\alpha}-\sum_{i=2}^{n} s_{i}\sum_{j=1}^{i-1} \frac{1}{j-\alpha}=0 \end{matrix}$$

With:

$$\begin{matrix} L=Log\left(n!\frac{\theta(\theta+\alpha)(\theta+2\alpha)...(\theta+(u-1)\alpha)}{\theta(\theta+1)...(\theta+n-1)}\prod_{j=1}^{n} \left[\frac{(1-\alpha)^{[j-1]}}{j!}\right]^{s_{j}}\frac{1}{s_{j}!}\right) \\ =const+\sum_{i=1}^{u-1} \log(\theta+i\alpha)-\sum_{j=1}^{n-1} \log(\theta+j)+\sum_{j=2}^{n} s_{j}\left(\sum_{i=1}^{j-1} \log(i-\alpha)\right) \end{matrix}$$

Where $const$is a value not depending on $\alpha$ or $\theta$.

Hoshino then solves the above equations by the Newton-Raphson method using the second derivates: $\frac{\partial^{2}L}{(\partial\theta)^{2}},\frac{\partial^{2}L}{(\partial\alpha)^{2}}$ and $\frac{\partial^{2}L}{\partial\theta\partial\alpha}$ with starting values:

$\theta=\frac{nuc-s_{1}(n-1)(2u+c)}{2s_{1}u+s_{1}c-nc}$ and $\alpha=\frac{\theta(s_{1}-n)+(n-1)s_{1}}{nu}$, where $c=\frac{s_{1}(s_{1}-1)}{s_{2}}$.

- **Slide negative binomial model:** This model assumes a slide negative binomial distribution for the population cell frequencies: $P(F_{k}=y)=\frac{\Gamma(\alpha+(y-1))}{\Gamma(\alpha)(y-1)!}\beta^{\alpha}(1-\beta)^{y-1}$ , where $\alpha$ and $\beta$ are the parameters of the gamma distribution that models the expected population cell frequency. The expected number of uniques in the population is then shown to be: $E(S_{1})=K\beta^{\alpha}$. To estimate the $\alpha$ and $\beta$ parameters, the following equations need to be solved numerically: $\begin{matrix} s_{1}=K\pi\left[\frac{\beta}{1-(1-\pi)(1-\beta)}\right]^{\alpha}\left[\frac{\alpha(1-\pi)(1-\beta)}{1-(1-\pi)(1-\beta)}+1\right] \\ s_{2}=K\frac{\alpha\beta^{\alpha}\pi^{2}(1-\beta)}{2\left[1-(1-\pi)(1-\beta)\right]^{\alpha+2}}\left[2-(1-\alpha)(1-\beta)(1-\pi)\right] \end{matrix}$

In our implementation we used the modified Shlosser estimate for $K$ described in [95]. This particular estimate was the most likely to result in convergence of the SNB model based on our simulations.

- **Mu-Argus:** This model has not been used in the context of population uniques estimation. However it can be used to calculate $\sum_{k} P(F_{k}=1|f_{k}=1)$, i.e., the expected number of sample uniques that are population uniques. The total number of population uniques can then be estimated from this quantity as is the case with the equivalence class model above, i.e. $S_{1}=\sum_{k} P(F_{k}=1|f_{k}=1)\frac{N}{n}$ It proposes a model with the assumption: $F_{k}/f_{k}\sim NB(f_{k},p_{k})+f_{k}$, this is the number of trials until $f_{k}$ successes occur with the probability of success being $p_{k}$. To calculate $P(F_{k}=1|f_{k}=1)$, one needs to estimate $p_{k}$. Benedetti proposed [65]: $\mathop{p_{k}}\limits^{\wedge} =\frac{f_{k}}{\mathop{F_{k}^{D}}\limits^{\wedge} }$ where $\mathop{F_{k}^{D}}\limits^{\wedge} =\sum_{i} w_{i}$ is the initial estimate for the population, where $w_{i}$ are the sampling weights. However, since we use simple random sampling we have $n=\mathop{p}\limits^{\wedge} _{k}N$. And the number of sample uniques that are population uniques becomes $\sum_{k} P(F_{k}=1|f_{k}=1)$ $=\sum_{f_{k}=1} \mathop{p_{k}}\limits^{\wedge} =s_{1}\pi$.
