# Supplementary material for: Estimating the re-identification risk of clinical data sets
Source: BMC Med Inform Decis Mak. 2012 Jul 9;12:66. doi: 10.1186/1472-6947-12-66 (PMC3583146; doi:10.1186/1472-6947-12-66)
Supplement: Additional file 2 — Appendix B. Detailed Results. [file 1472-6947-12-66-S2.pdf]

Appendix B: Detailed Results

In this appendix we provide the detailed results for all of the estimators under all conditions. The graphs show the median and inter-quartile range of the relative bias for the 1000 simulation runs. Note that the FARS and Adult data did not meet the criteria for high uniqueness in the original data set, and therefore these two data sets are missing in the high uniqueness figures below. The "pi" in the figures refers to the sampling fraction.

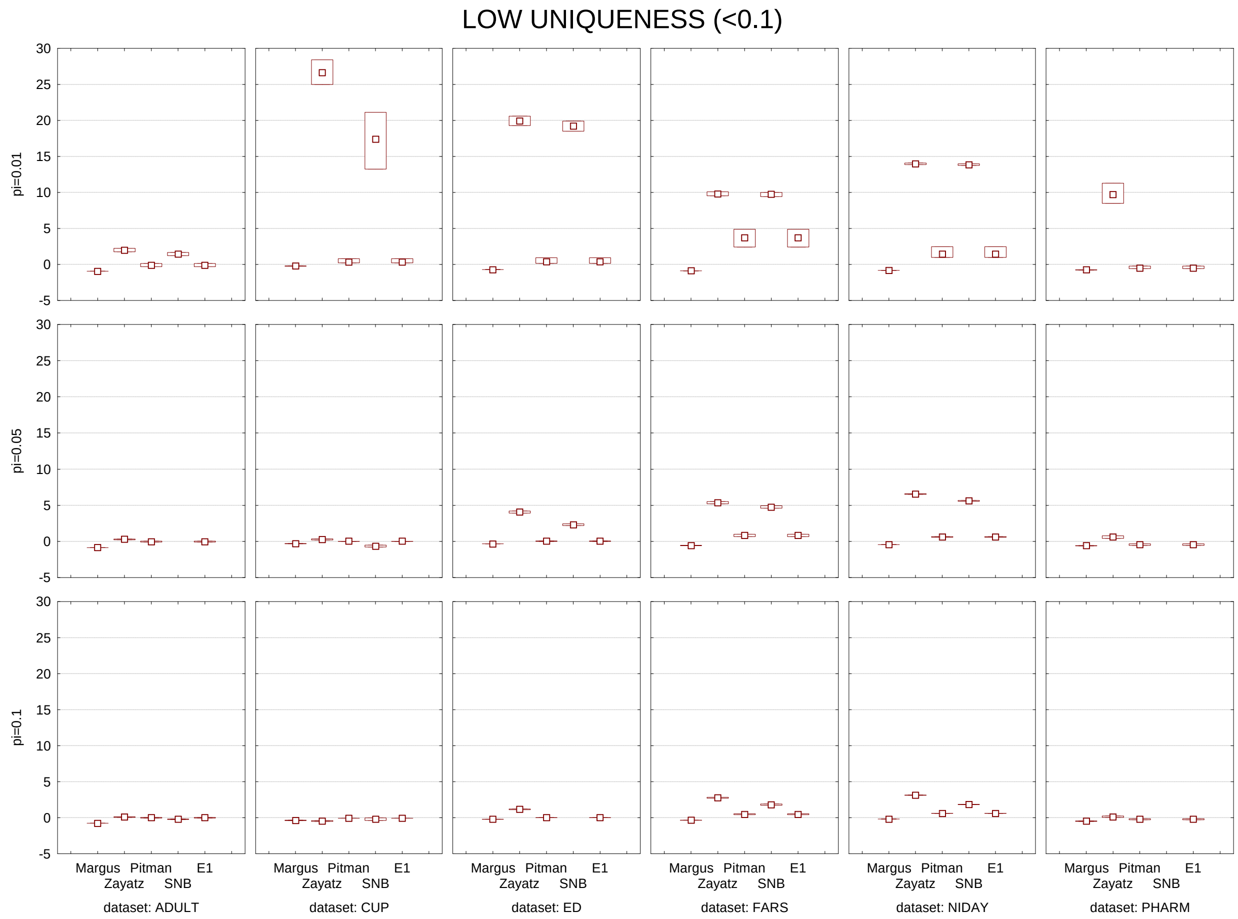

Figure B.1: The median relative error and inter-quartile range for the six data sets for the low uniqueness population at the lowest sampling fractions.

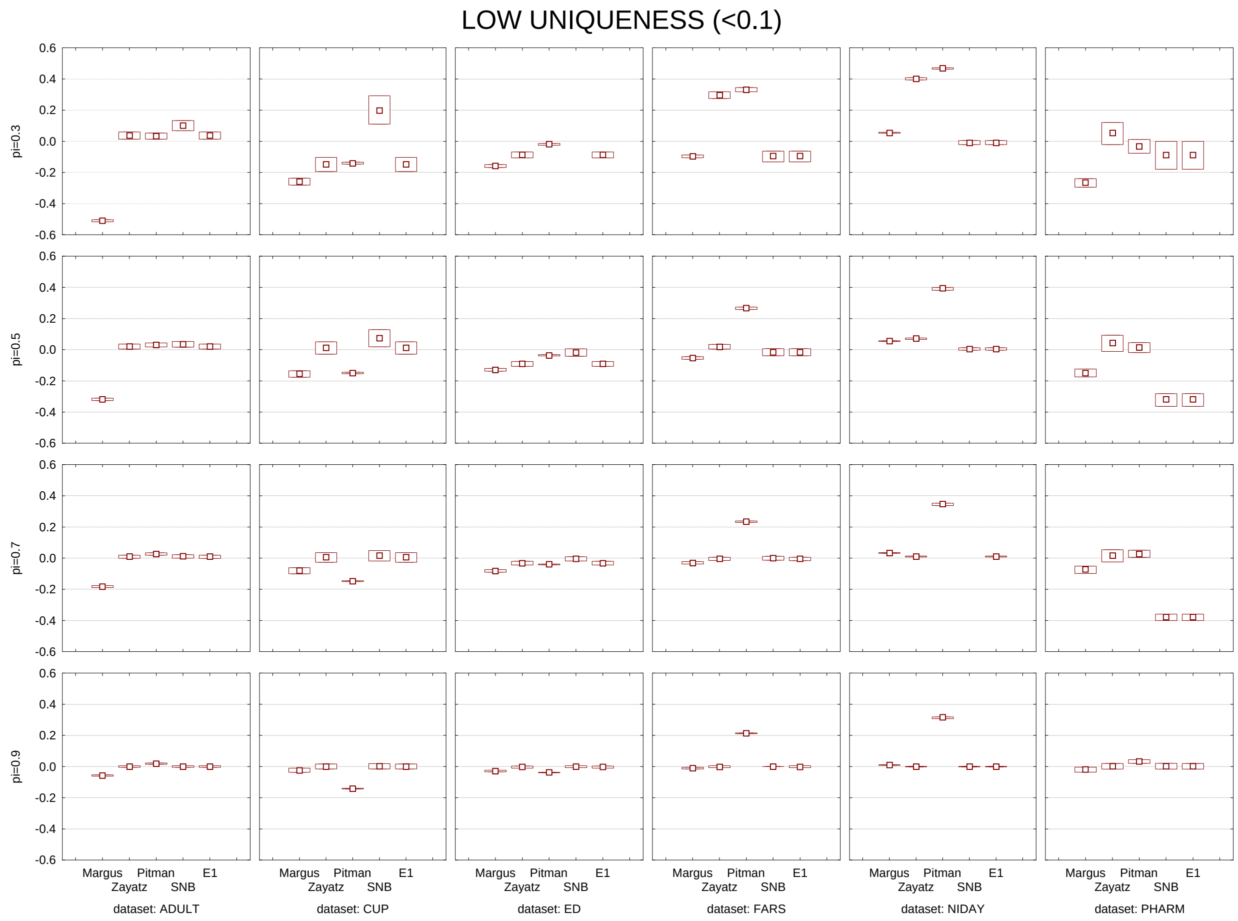

Figure B.2: The median relative error and inter-quartile range for the six data sets for the low uniqueness population.

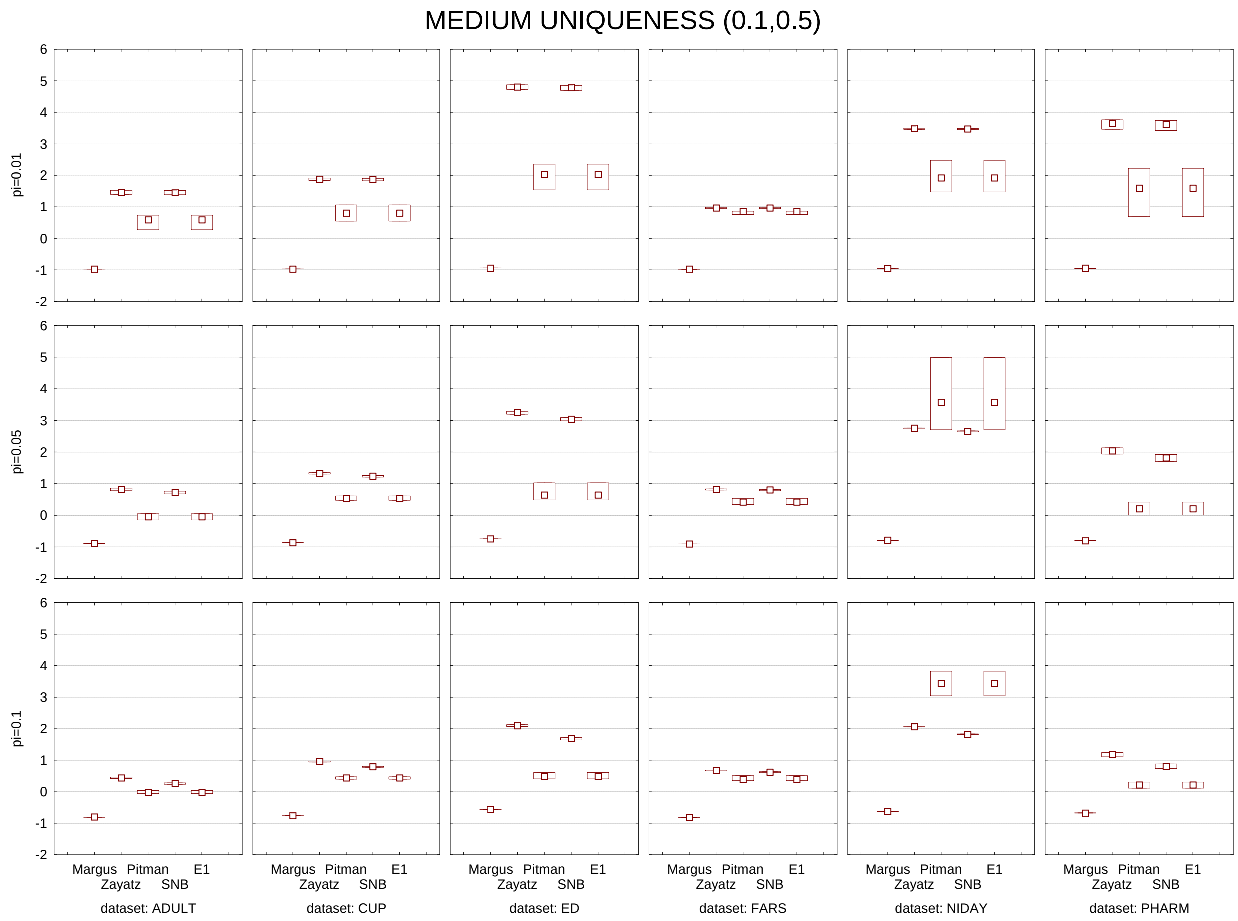

Figure B.3: The median relative error and inter-quartile range for the six data sets for the medium uniqueness population at the lowest sampling fractions.

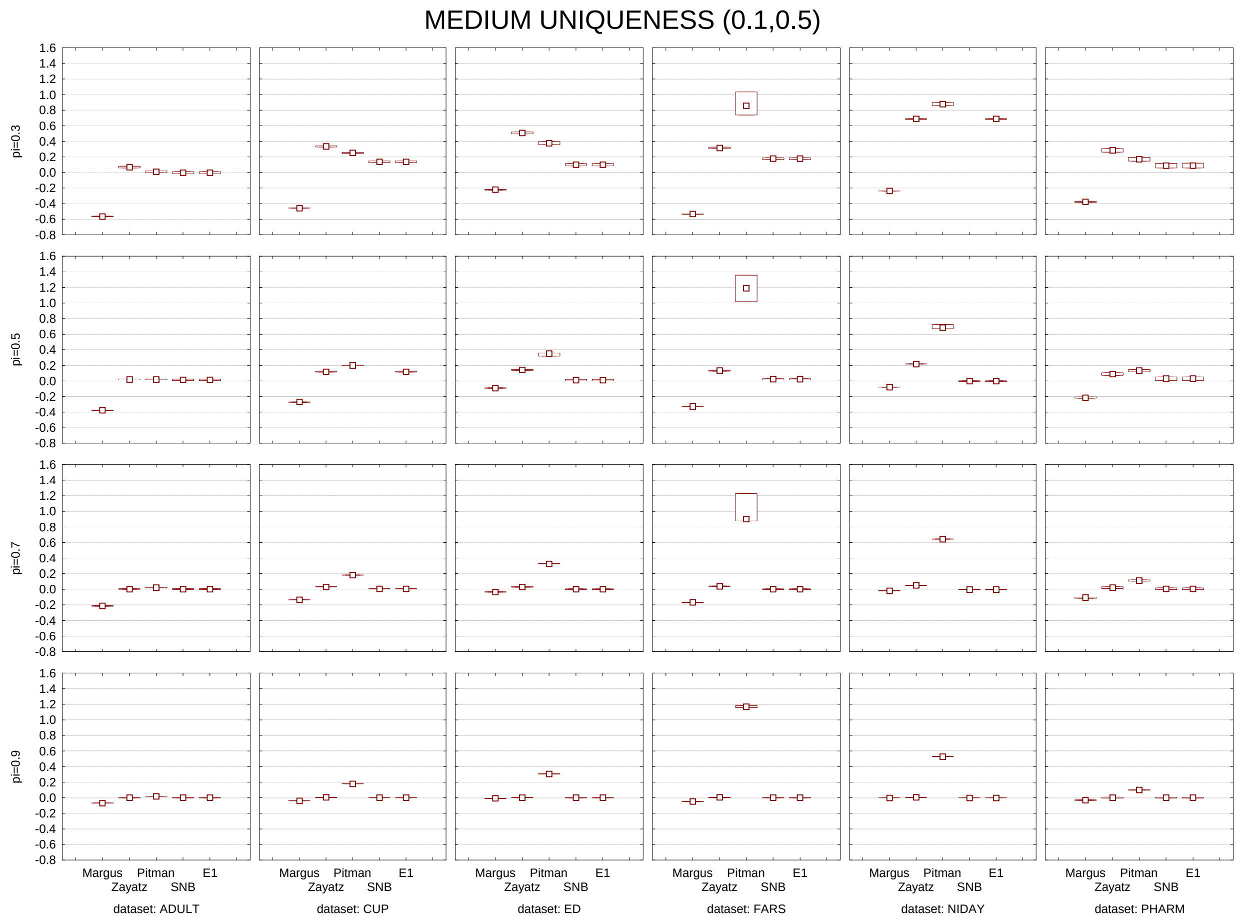

Figure B.4: The median relative error and inter-quartile range for the six data sets for the medium uniqueness population.

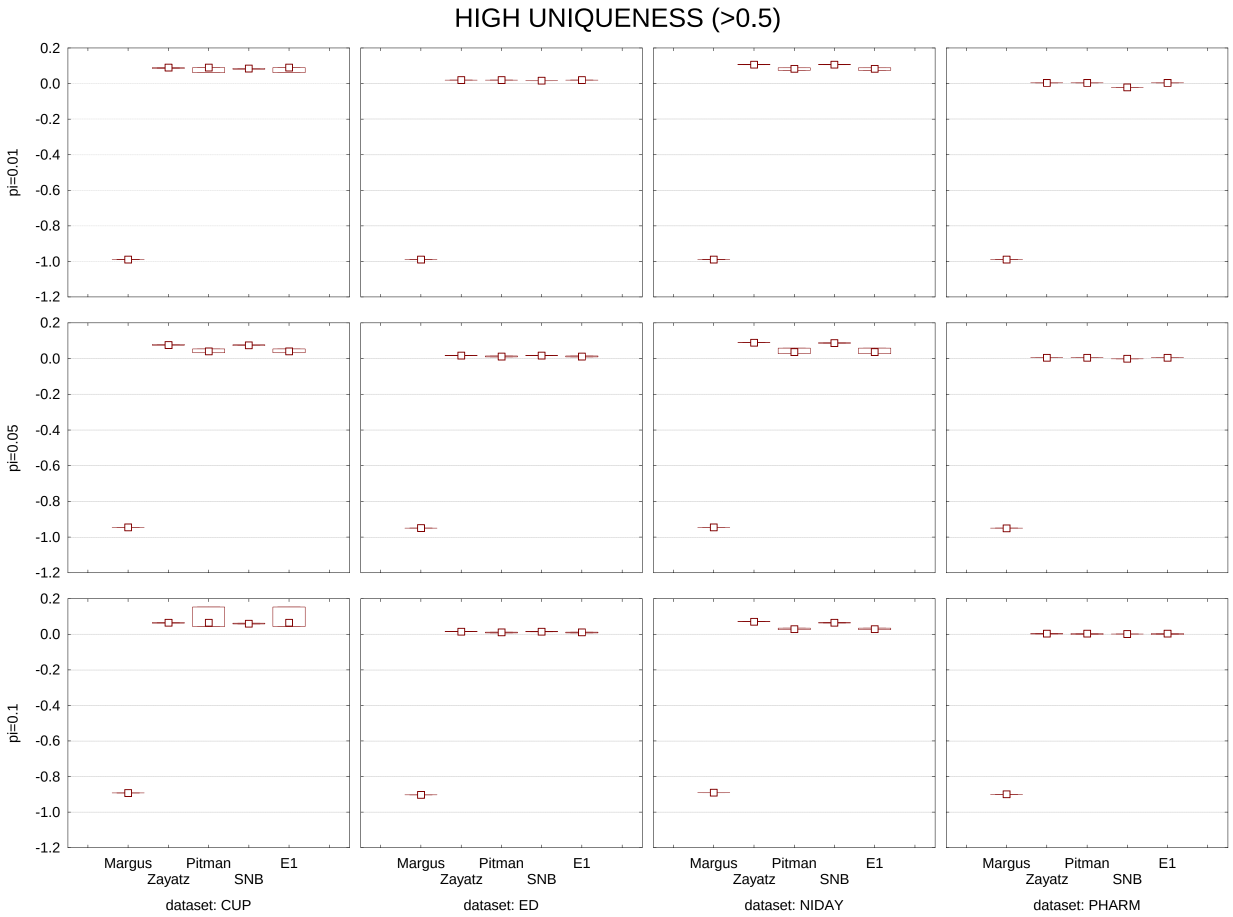

Figure B.5: The median relative error and inter-quartile range for four of the data sets for the high uniqueness population at the lowest sampling fractions.

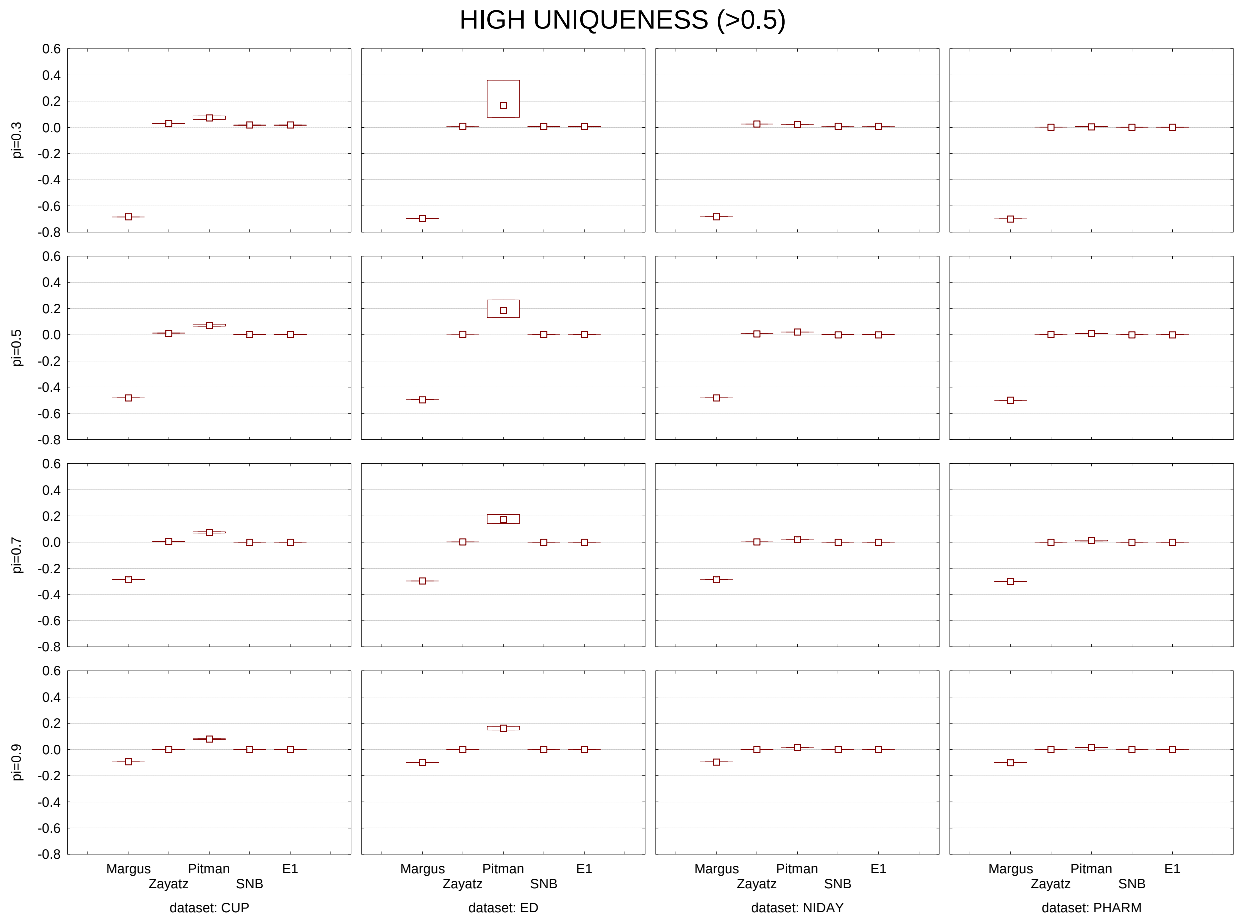

Figure B.6: The median relative error and inter-quartile range for four of the data sets for the high uniqueness population.
